# Grammatical Role Parallelism Influences Ambiguous Pronoun Resolution in German

**DOI:** 10.3389/fpsyg.2017.01205

**Published:** 2017-07-25

**Authors:** Antje Sauermann, Natalia Gagarina

**Affiliations:** Leibniz-Zentrum Allgemeine Sprachwissenschaft Berlin, Germany

**Keywords:** pronoun resolution, parallelism, grammatical role, word order, German

## Abstract

Previous research on pronoun resolution in German revealed that personal pronouns in German tend to refer to the subject or topic antecedents, however, these results are based on studies involving subject personal pronouns. We report a visual world eye-tracking study that investigated the impact of the word order and grammatical role parallelism on the online comprehension of pronouns in German-speaking adults. Word order of the antecedents and parallelism by the grammatical role of the anaphor was modified in the study. The results show that parallelism of the grammatical role had an early and strong effect on the processing of the pronoun, with subject anaphors being resolved to subject antecedents and object anaphors to object antecedents, regardless of the word order (information status) of the antecedents. Our results demonstrate that personal pronouns may not in general be associated with the subject or topic of a sentence but that their resolution is modulated by additional factors such as the grammatical role. Further studies are required to investigate whether parallelism also affects offline antecedent choices.

## Introduction

Pronoun resolution has “traditionally" been examined separately by linguists and psychologists. Yet, more recently both areas have come closer together. This lead to the insight that anaphor/pronoun resolution is influenced by several factors. More importantly, the eye-tracking technique in the visual world paradigm has been shown to be particularly useful to examine pronoun/anaphor resolution during online processing. In this paradigm, an auditory stimulus is presented together with visual stimuli (e.g., two pictures) with the eye-movements on the pictures reflecting pronoun resolution preferences. Crucially, the online technique may reveal factors that influence pronouns resolution during online processing that may not be detected when offline techniques, e.g., judgments, are used (Schumacher et al., 2017). We used the visual world paradigm to investigate the impact of grammatical role parallelism which may likely to occur during online processing, i.e., exactly when the pronoun is processed (Smyth, 1994).

### Factors Influencing Pronoun Resolution

The factors influencing pronoun resolution have been intensively investigated in the last few decades. Pronoun resolution usually involves a process wherein an anaphor [e.g., the pronoun “he” in (1)] is associated with an antecedent in the previous context (e.g., “Goofy”).

(1) Goofy greets Donald. He...

Pronoun resolution requires the integration of different sources of information (e.g., Smyth, 1994; Arnold et al., 2000; Kehler et al., 2008; Schumacher et al., 2015, 2016, 2017). First, syntactic factors, e.g., gender and number agreement and binding principles, constrain pronoun resolution. Second, different strategies may influence pronoun resolution in ambiguous contexts like (1), where the personal pronoun *he* may refer to *Goofy* or *Donald*, but participants usually prefer *Goofy*.

Resolution preferences in ambiguous contexts are influenced by the information status of the antecedent, i.e., personal pronouns refer to the most salient referents (e.g., Gundel et al., 1993; Ariel, 2001). The salience of an antecedent may be induced by several factors, among them its grammatical role (e.g., Crawley et al., 1990; Stevenson et al., 1994; Grosz et al., 1995; Bosch et al., 2007), thematic role (e.g., Schumacher et al., 2015, 2016, 2017), sentence position (Gernsbacher and Hargreaves, 1989; Stevenson et al., 1994) or information and discourse status (e.g., Grosz et al., 1995; Bosch et al., 2003; Bosch and Umbach, 2007; Kaiser and Trueswell, 2008; Colonna et al., 2012; Ellert, 2013).

The impact of one or the other factor from this list is usually difficult to disentangle. In addition, these factors interact with parallelism (e.g., Smyth, 1994; Stevenson et al., 1995; Chambers and Smyth, 1998), verb semantics (e.g., Grober et al., 1978; Schumacher et al., 2015, 2016, 2017), discourse relations (e.g., Grober et al., 1978; Kehler et al., 2008; see also Kaiser, 2011) and the type of referring expression realizing the anaphor (e.g., Gundel et al., 1993; Ariel, 2001; Bosch et al., 2003; Bosch et al., 2007; Kaiser and Trueswell, 2008; Schumacher et al., 2015).

Accordingly, different factors may be responsible for the subject preference of the anaphoric pronouns in the subject position, like, e.g., *he* in (1). These factors are difficult to tease apart because their features overlap and they make similar predictions. That is, *Goofy* may be the preferred antecedent because it is the subject and topic or because it shares the grammatical role and initial sentence position of the anaphor *he*. Languages with a more flexible word order than English, for instance German, provide a means to disentangle these factors. The goal of our study is to examine the impact of the word order of the antecedent sentence and grammatical role parallelism on pronoun resolution in German by using the visual world paradigm. First, we will review the research on pronoun resolution in German and then we will present our study. A discussion and conclusion will close the paper.

### Pronoun Resolution in German

German is a language with a relatively flexible word order, that allows besides the canonical SVO word order (2a) also the non-canonical OVS word order (2b). Word order variation has been linked to the information structure factors, in that the sentence-initial position is usually seen as a topic position (e.g., Frey, 2006). Thus, in SVO sentences the subject is seen as the topic and in OVS sentences the object.

(2)a) Der Mann grüßt den Jungen. Er/Der...
  the_NOM_^1^ man greets the_ACC_ boy he_PRO_/he_DEM_
(2)b) Den Mann grüßt der Junge. Er/Der...
  the_ACC_ man greets the_NOM_ boy he_PRO_/he_DEM_

Studies on pronoun resolution in German have mainly dealt with personal pronouns like *er* (“he_PRO_”) and demonstrative pronouns (d-pronouns) like *der* (“he_DEM_”) in contexts with the SVO (2a) or OVS (2b) word order of the antecedents.

Research on SVO sentences has shown that *er* (“he_PRO_”) is usually resolved to *der Mann* (“the man”) and *der* (“he_DEM_”) to *den Jungen* (“the boy”) (e.g., Bosch et al., 2003; Bouma and Hopp, 2007; Colonna et al., 2012; Schumacher et al., 2015, 2016, 2017; but see Bosch et al., 2007), but with a stronger preference for d-pronouns compared to pronouns (e.g., Bosch et al., 2003, 2007; Bouma and Hopp, 2007; Schumacher et al., 2015, 2017).

For OVS sentences the pattern is less coherent. While d-pronouns show a preference to refer to objects (Schumacher et al., 2015, 2017), personal pronouns prefer subjects (e.g., Bouma and Hopp, 2007; Schumacher et al., 2017) or show no preference (Bosch and Umbach, 2007; Bosch et al., 2007; Colonna et al., 2012; Schumacher et al., 2015). This variation in the pronoun resolution may be due to differences in the experimental material and settings, i.e., in the use of verbs (e.g., Schumacher et al., 2015, 2016, 2017), in the discourse relations between both sentences (e.g., Kaiser, 2011) or in the presence (or absence) of a preceding context which licenses the non-canonical word order (cf., Schumacher et al., 2017). In addition, differences in the methods used, especially in the use of offline or online experiments, may have led to incoherent results (cf., Schumacher et al., 2017; see also Bosch et al., 2007).

Despite this variation in the results, the majority of studies agree that subject personal pronouns usually refer to the subject (e.g., Bosch et al., 2007) or topic antecedent and indicate topic continuity (Bosch et al., 2003; Bosch and Umbach, 2007; Schumacher et al., 2016) whereas d-pronouns refer to non-subjects (e.g., Bosch et al., 2007) or less-topical referents and indicate a topic shift (Bosch et al., 2003; Bosch and Umbach, 2007; Schumacher et al., 2016). In addition, thematic status, subject status and information status have separate effects on pronoun resolution, which can be revealed when different constructions are investigated (e.g., Ellert, 2013; Schumacher et al., 2015, 2016).

While the resolution of subject pronouns has been explored much more intensively, only a few studies have investigated the resolution of pronouns with other grammatical roles, e.g., object pronouns. In these cases (e.g., Crawley et al., 1990; Smyth, 1994; Stevenson et al., 1994, 1995; Chambers and Smyth, 1998; Wolf et al., 2004; Kehler et al., 2008), researchers mainly examined English and used parallel structures like those in (3).

(3) Goofy greets Donald, and Daisy hugs him.

For these structures, some studies showed that the object pronoun *him* was associated with the object antecedent *Donald* (e.g., Smyth, 1994; Stevenson et al., 1995; Chambers and Smyth, 1998; Wolf et al., 2004; Kehler et al., 2008). However, other studies failed to provide evidence for this preference (e.g., Crawley et al., 1990; Stevenson et al., 1994), indicating that the resolution is influenced by additional factors like verb semantics (e.g., Grober et al., 1978) or discourse relations (e.g., Kehler et al., 2008).

Crucially, the previous studies on English examined structures with three types of parallelism: first, *grammatical role parallelism*, with respect to the grammatical role (i.e., *him* and *Donald* are both the object), a second, *positional parallelism*, with respect to the position (*him* and *Donald* both occur in the sentence-final position), and a third, *structural parallelism*, with respect to the similar structures of the antecedent and anaphora sentences. Especially, the third type has been shown to have a strong impact on sentence processing (e.g., Sheldon, 1974; Frazier et al., 1984; Carlson, 2001; Callahan et al., 2010; Poirier et al., 2012 on English; Weskott, 2003; Knoeferle and Crocker, 2009 on German).

With respect to anaphor resolution, Smyth (1994) and Stevenson et al. (1995) tried to disentangle these factors. Stevenson et al. (1995) provide evidence against a “pure” position effect in the resolution of subject pronouns. Smyth (1994) showed that parallelism of the grammatical role had a strong impact on pronoun resolution, but structural parallelism also had an effect. However, neither study tested structures in the non-canonical word order, which may provide a clearer way to untangle the position effect and parallelism with respect to the grammatical role.

### The Present Study

**FIGURE 1:**
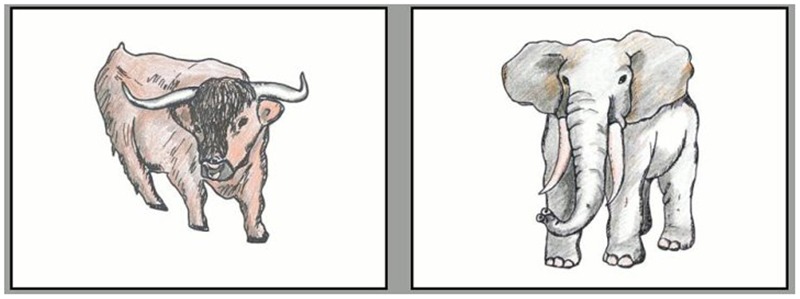
Sample pictures accompanying the trials presented in (4).

We report on a visual world eye-tracking study that aimed to examine the impact of the word order and grammatical role parallelism on the online comprehension of personal pronouns. In the visual world paradigm the linguistic material [see (4)] is presented together with pictures of the possible antecedents (**Figure 1**), with the looks to the pictures of the antecedents reflecting pronoun resolution preferences during online processing.

We presented the antecedents in the canonical SVO (4a, 4b) or the non-canonical (4c, 4d) word order, with the case morphology of the determiners of the noun phrases (NPs) indicating grammatical role and word order. Grammatical role parallelism effects were tested by presenting the anaphoric pronoun either as the subject (4a, 4c) or as the object (4b, 4d).

**Table:** 

(4)	Der Bulle und der Elefant spielen zusammen Verstecken im Wald.				
	“The bull and the elephant are playing hide and seek in the forest.”					
a)	Der	Bull	sieht	den	Elefanten.	Er …	ist	traurig.		(SVO, sbj)
	the_NOM_	bull	sees	the_ACC_	elephant	he_NOM_	is	sad	
	“The bull sees the elephant. He … is sad.”					
b)	Der	Bull	sieht	den	Elefanten.	Ihn …	trifft	der	Blitz.	(SVO, obj)
	the_NOM_	bull	sees	the_ACC_	elephant	he_ACC_	hits	the_NOM_	lightning
	“The bull sees the elephant. Him … the lightning hits.”						
c)	Den	Bullen	sieht	der	Elefant.	Er …	ist	traurig.		(OVS, sbj)
	the_ACC_	bull	sees	the_NOM_	elephant	he_NOM_	is	sad
	“The bull, the elephant sees. He … is sad.”						
d)	Den	Bullen	sieht	der	Elefant.	Ihn …	trifft	der	Blitz.	(OVS, obj)
	the_ACC_	bull	sees	the_NOM_	elephant	he_ACC_	hits	the_NOM_	lightning
	“The bull, the elephant sees. Him … the lightning hits.”						

This design, i.e., the comparison of subject and object anaphors, allows us to test the prediction that personal pronouns in general refer to the subject in the preceding antecedent sentence. If this is the case, we expect a higher proportion of looks to the picture of the subject antecedent compared to the object antecedent for both subject and object anaphora in the canonical word order (4a, 4b). With respect to the non-canonical word order (4c, 4d), the eye-tracking study by Schumacher et al. (2017) found a subject preference for subject anaphora (regardless of the word order) for accusative verbs, whereas offline studies revealed a less coherent pattern (e.g., Bosch et al., 2007; Schumacher et al., 2015). Given that our study is also an online study, we expect a subject preference for subject anaphora in our data.

If parallelism of a grammatical role plays a strong role during online processing, subject pronouns should be resolved to subject antecedents and object pronouns to object antecedents regardless of the word order of the antecedents. However, additional factors, e.g., positional and structural parallelism, may also play a role.

That is, if positional parallelism influences pronoun resolution, both subject and object pronouns should be resolved to the first mentioned antecedent in our study, i.e., to the subject in SVO sentences and the object in OVS sentences. That is, we expect an interaction between Pronoun Type and Word Order on the looks to the subject antecedents.

If structural parallelism influences pronoun resolution, we expect that subject pronouns are resolved to subject antecedents in SVO sentences (condition a) and object pronouns to object antecedents in OVS sentences (condition d). In the conditions without structural parallelism (conditions 4b and 4c), we expect less clear resolution preferences.

## Materials and Methods

### Design and Materials

The experiment employed a 2 × 2 repeated-measures design with Word Order (SVO vs. OVS) and (the grammatical role of the) Pronoun (“subject” (sbj) vs. “object” (obj)) as independent variables and the eye-movements, i.e., the proportion of looks to the subject of the SVO or OVS sentence, as dependent variable.

The experimental trials [see (4)] started with a sentence introducing the two referents, which was followed by an antecedent SVO or OVS word order sentence. The grammatical role of the antecedents was indicated by case marking of the first and second NP: the determiner *der* indicated nominative case and subject status, and the determiner *den* indicated accusative case and object status. The antecedent sentence was followed by a second sentence with the subject pronoun *er* (“he”) or object pronoun *ihn* (“him”) in the initial position. The pronoun sentence was interrupted by a pause of 500 ms after the offset of the pronoun.

The verbal stimulus was accompanied by two pictures depicting the two animals mentioned in the discourse (see **Figure 1** above). The pictures had a size of 440 pixels × 330 pixels and were placed horizontally at the left or right side of the screen, separated from each other by approximately 25 pixels.

Four experimental items (animal pairs) were created (see Supplementary Material for the complete list of the items). For each item two versions of the trials were created controlling for the effects of order of mention and positioning of the pictures. That is, for each trial we created an alternative version wherein the elephant was the first NP in the lead-in and antecedent sentence and the picture was presented on the left side. Each participant saw all four conditions of an item. The reason for this experimental design and the low number of items was that the experiment was also run with bilingual preschoolers, who should know the meanings of the verbs used.

In addition two practice trials and eight filler sentences were created. Each trial was accompanied by two pictures of two animals. Practice trials consisted of an introduction sentence and a transitive sentence, similar to the SVO condition of the experimental trials. However, these sentences were not followed by a pronoun sentence. Fillers were SVO sentences that mentioned the two animals depicted.

### Procedure

Participants were seated in front of a 15″ laptop on which the experimental sentences were presented. The experiment involved a looking-while-listening task. That is, participants were not instructed to perform a specific task but only to listen to short stories that were accompanied by two pictures.

Each experimental session began with a 5-point calibration procedure to adjust the eye-tracking system. The experiment started with two practice sentences. Each participant saw 16 experimental trials, with a filler sentence being shown after every two experimental trials. Participants were tested using four test lists that were created to control for the positioning of the pictures and the order of the mention of the animals.

Data were recorded using a portable Tobii X2-60 Compact eye-tracking system (Tobii Technology AB, Sweden), which was attached to the laptop. Eye-movements were sampled with a tracking rate of 60 Hz, approximately every 16 ms.

### Data Treatment and Analyses

The eye-movement recordings were based on the gazes as determined and pre-processed by the Tobii Studio software (Version 3.2.2, Tobii Technology AB, Sweden). Trials with more than 50 percent track loss (looks off screen) were excluded from further analysis (1%).

The eye-movement data was aggregated in 50 ms bins and analyzed in twelve 250 ms time windows from the onset of the pronoun until the end of the sentence. For the statistical analyses, we calculated the empirical logit for the looks to the picture of the subject antecedent, aggregating over items (cf., Barr, 2008). Looks to the subject antecedent picture were almost complementary to looks to the object antecedent picture because looks to neither of the pictures were rare (2%).

The lme4 package (version 1.1-12; Bates et al., 2015) was used to calculate linear mixed-effects models to assess the fixed effects of Word Order, Pronoun, Time and their interactions, and the random effect of Participants on the empirical logit of the looks to the target picture. The models included the weightings recommended for empirical logit analyses (Barr, 2008). The specification of the random effects of Participants considered the slope adjustment for Pronoun and Word Order and their interaction (cf., Barr et al., 2013). Time was not considered for the slope adjustment because models that included Time for slope adjustment led to convergence errors.

The contrast codings of predictors and Word Order (SVO: +1, OVS: -1) and Pronoun (er: +1, ihn: -1) and their interaction resembled those of traditional ANOVA analyses. The continuous predictor Time captured the five time (50 ms) bins that were analyzed in each 250 ms time window.

### Participants

Eighteen students of the Humboldt University Berlin participated in the study (13 women, mean age: 27 years). They were monolingual native speakers of German and had normal or corrected to normal vision.

This study was carried out in accordance with the recommendations of the Declaration of Helsinki with written informed consent from all subjects. All subjects gave written informed consent prior to participation in accordance with the Declaration of Helsinki. The protocol was approved by the German Linguistic Society (Deutsche Gesellschaft für Sprachwissenschaft, DGfS)

## Results

**FIGURE 2:**
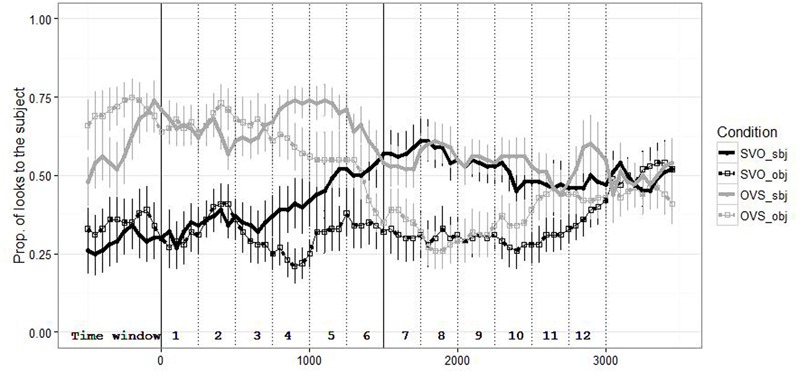
Mean proportion (with SE) of looks to the subject antecedent depending on Word Order and Pronoun. Standard errors (SE) exclude between-participant variance (Cousineau, 2005) and were normalized using Morey’s (2008) correction. Note that looks to the object antecedent were complementary to the looks to the subject.

**Figure 2** shows the mean proportion of looks to the subject calculated on 50 ms time bins starting with the offset of the antecedent sentence (SVO vs. OVS). The proportions of looks following SVO sentences are shown in black color and those following OVS sentences in gray. Solid lines indicate trials with the subject pronoun and dotted lines those with the object pronoun. The solid vertical lines indicate the onset of the pronoun (*er* or *ihn*) and the onset of the continuation of the sentence. Dotted vertical lines indicate the time windows.

**Table 1** lists the intercept (*b*) and *t*-values (*t*) for the fixed effects of the models in each time window. The models revealed a significant effect of word order in the first five time windows (until 1250 ms), resulting from fewer looks to the subject following SVO sentences whereas there were more looks to the subject following OVS sentences. The effect gradually declined in the fifth and sixth time windows, as indicated by the Pronoun–Time interaction, and did not occur in the subsequent time windows. We propose that this eye-movement pattern reflects the looks to the last-mentioned referent of the transitive sentence, i.e., the subject in OVS and the object in SVO sentences.

**Table 1:** Fixed effects of the models predicting the looks to the subject picture (significant values at α = 0.05, |*t*| ≥ 2 are indicated in bold).

	1 (0–250)		2 (250–500)		3 (500–750)		4 (750–1000)		5 (1000–1250)		6 (1250–1500)	
	*b*	*t*	*b*	*t*	*b*	*t*	*b*	*t*	*b*	*t*	*b*	*t*
Intercept	-0.104	-0.681	-0.055	-0.294	0.008	0.049	-0.066	-0.468	-0.084	-0.544	0.076	0.470
Pronoun	0.081	0.679	0.012	0.117	-0.045	-0.333	0.227	1.643	**0.430**	**2.789**	**0.397**	**2.151**
Word order	-**0.887**	-**4.956**	-**0.706**	-**3.809**	-**0.690**	-**3.600**	-**0.811**	-**4.212**	-**0.714**	-**3.126**	-0.367	-1.998
Time	0.000	0.283	**0.001**	**2.416**	-0.000	-1.015	0.000	0.179	**0.001**	**2.195**	-**0.001**	-**4.043**
Pron × WO^∗^	-0.046	-0.302	0.011	0.130	0.126	0.996	0.057	0.459	0.009	0.054	0.048	0.280
Pron × Time	0.000	0.520	-**0.001**	-**2.808**	0.001	1.870	**0.001**	**2.863**	0.000	0.083	0.000	1.283
WO × Time	0.001	1.645	0.000	1.036	-**0.001**	-**2.047**	0.000	0.423	**0.001**	**3.522**	**0.001**	**3.234**
Pron × WO × Time	0.000	0.789	0.000	0.647	-0.000	-0.209	-0.000	-0.632	0.000	0.762	0.000	0.335

	**7 (1500–1750)**		**8 (1750–2000)**		**9 (2000–2250)**		**10 (2250–2500)**		**11 (2500–2750)**		**12 (2750–3000)**	
	***b***	***t***	***b***	***t***	***b***	***t***	***b***	***t***	***b***	***t***	***b***	***t***

Intercept	-0.271	-1.347	-0.177	-1.574	-0.346	-2.929	-0.315	-2.387	-0.340	-2.384	-0.291	-2.053
Pronoun	**0.582**	**2.724**	**0.715**	**4.311**	**0.570**	**3.957**	**0.470**	**2.929**	0.296	1.567	0.186	1.159
Word order	-0.086	-0.500	0.038	0.264	-0.001	-0.006	-0.029	-0.221	-0.230	-1.611	-0.133	-0.899
Time	-0.000	-1.226	-0.001	-1.538	0.000	1.057	-0.000	-1.397	-0.000	-0.124	**0.001**	**2.844**
Pron × WO	0.128	0.926	0.059	0.463	-0.032	-0.307	0.017	0.171	0.041	0.314	0.106	0.779
Pron × Time	0.000	0.732	-0.000	-1.221	-0.000	-0.901	-0.000	-0.224	-**0.001**	-**2.486**	0.001	1.780
WO × Time	0.000	0.661	0.000	0.270	-0.000	-1.124	-**0.001**	-**2.788**	0.000	0.878	0.000	0.246
Pron × WO × Time	0.000	0.453	-0.001	-1.665	0.000	0.471	-0.000	-0.703	0.001	1.710	-**0.001**	-**3.038**

*^∗^Pron, pronoun; WO, word order.*

The pronoun type influenced the eye-movement from around 750 ms (starting with the fourth time window), as a significant interaction between Pronoun and Time revealed.^2^ This interaction indicates that the difference between subject and object anaphora increased with time. That is, looks to the subject antecedent gradually increased after subject anaphora (solid gray and black lines) and gradually decreased after object anaphora (dotted gray and black lines) during the time interval from 750–1000 ms, i.e., in the fourth time window. Notably this effect occurred in both word orders. The main effect of Pronoun was fully established in the fifth time window (from 1000 ms) and continued until the tenth time window (until 2500 ms). In the eleventh time window (2500–2750 ms), the Pronoun effect gradually disappeared, as indicated by the interaction between Pronoun and Time. In the final time window (2750–3000 ms), there was a significant interaction between Pronoun, Word Order and Time as well as a main effect of Time. *Post hoc* comparisons assessing the impact of Time and Word Order for each pronoun type revealed a significant effect of Time reflecting a gradual increase in the eye-movements for subject anaphora (*b* = 0.002, *t* = 3.431, especially in OVS trials) but no change in the eye-movements for object anaphora (*b* = 0.000, *t* = 0.691). However, given that this time window was at the end of the trial and the eye-movements in all conditions centered around chance-level, the effects in the last two time windows are difficult to interpret.

## Discussion

The eye-tracking study examined the effects of word order and grammatical role parallelism on anaphora resolution in adult German. Antecedent sentences with SVO and OVS word order and sentences with subject vs. object pronominal anaphora composed four contexts which were investigated [see examples in (4)].

The results showed that grammatical role parallelism influenced online pronoun resolution in both word orders. This was reflected by the eye-movements starting around 750 ms after pronoun onset such that looks to the subject antecedent increased in subject anaphor trials compared to object anaphor trials in both word orders. Given that looks to subject and object antecedents were complementary, this also reflects that looks to the object increased after object anaphora trials compared to subject anaphora trials. This pattern occurred even before the anaphor sentence was continued, suggesting that it cannot be attributed to the different sentence continuations for subject and object anaphora.

Importantly, this effect of the pronoun type was not influenced by an interaction with word order. This suggest that the resolution preferences resulted from parallelism of the grammatical role and were not restricted to a particular position of an antecedent or to similarities of the syntactic structure of the antecedent and anaphor sentence. Thus, the pronoun resolution in our study was not influenced by positional or structural parallelism.

Nevertheless, the eye-movements were initially also influenced by the word order of the sentence, reflecting that participants looked at the last-mentioned antecedent. This effect did not interact with the grammatical role of the anaphora and apparently resulted from the experimental design. In addition, in later time windows when the sentence continued, the word order effect gradually decreased and did not affect eye-movements.

Our results strongly indicate that the grammatical role of the anaphor influences its resolution shortly after the pronoun is heard and processed and even before the anaphor sentence is continued. Indeed, the time window wherein the impact of the anaphora occurred in our study corroborates the results of the visual world study by Schumacher et al. (2017), who found an impact of the demonstrative and personal pronouns on their resolution in accusative verb sentences only slightly earlier (400–600 ms after pronoun onset). This suggests that not only the type of the referring expression but also the grammatical role impacts online pronoun resolution.

The early effect of the grammatical role of the pronoun corresponds to the proposal by Smyth (1994). Similar to previous research concluding that pronoun resolution starts immediately after the pronoun is heard (e.g., Ehrlich and Rayner, 1983; see also Arnold et al., 2000; Schumacher et al., 2016, 2017), Smyth suggests that parallelism influences pronoun resolution in terms of a feature match process whereby antecedents are selected on the basis of the features they share with the anaphora – in our case, grammatical role features. In our study, this effect was not restricted to structures in which the antecedent sentence and the anaphor sentence share the same word order, i.e., positional parallelism. This differs from the studies demonstrating a strong impact of positional (or structural) parallelism on sentence processing (e.g., Sheldon, 1974; Frazier et al., 1984; Carlson, 2001; Weskott, 2003; Knoeferle and Crocker, 2009; Callahan et al., 2010), including pronoun resolution (e.g., Smyth, 1994; Poirier et al., 2012). This difference may result from the materials (e.g., the lack of a conjunction or the pause within the pronoun sentence in our study) or the methodology used.

While our data also show a stable effect of the pronoun, reflecting the grammatical role parallelism effect, until 2250 ms after the pronoun onset, this did not influence eye-movements in the last two time regions. The lack of the effect in these time regions may merely result from the fact that they appear at the very end of the trial. Alternatively, it may indicate that grammatical role parallelism effects may be weaker during later processing or influenced by the predicate of an anaphora sentence. This instability with respect to the resolution preferences was also found in Schumacher et al.’s (2017) research and was evidenced by the differences between their online and offline study. While their online eye-tracking study revealed a subject preference for subject personal pronouns in SVO and OVS sentences (Schumacher et al., 2017), their offline rating study showed the subject preference in SVO sentences only (Schumacher et al., 2015).

Given that we did not test offline antecedent choice, we can only draw cautious predictions about the offline interpretation of the subject and object personal pronouns in our data. Nevertheless, our results reflect a stable effect of the grammatical role. This suggests that personal pronouns in the initial position in a sentence are not generally – irrespective of the other factors – resolved to subjects but that their resolution preferences are also modulated by the grammatical role parallelism of a pronominal anaphora and its antecedent. This corresponds to previous work (e.g., Bosch and Umbach, 2007; Bosch et al., 2007; Schumacher et al., 2015, 2017) demonstrating that (subject) personal pronouns show weaker antecedent preferences compared to demonstrative pronouns.

Notably, visual inspection of the eye-movement plot may indicate that the impact of the grammatical role was somewhat stronger for object anaphora compared to subject anaphora because the eye-movements for subject anaphora were closer to the 50% chance level. This apparently weak preference for subject anaphora also corresponds to the differences between personal and demonstrative pronouns mentioned above. However, this does not explain why object anaphora show a clearer preference for object antecedents.

It might be that hearers rely more on parallelism when the object pronoun follows the less frequent and more marked OVS word order in the antecedent sentence. Following the SVO sentence, the OVS sentences with an object anaphor may indicate a topic shift with the object as the new topic. Following the OVS antecedent sentence, structural parallelism with the OVS sentence may facilitate OVS sentence comprehension in general (cf., Weskott, 2003; Knoeferle and Crocker, 2009) and thus may enhance grammatical role parallelism effects. If this is the case, parallelism effects may interact with information structure factors. However, further research that considers corpus data and antecedent choice tasks is needed to clarify the differences between subject and object anaphora.

In general, our study underlines the importance of considering different empirical methods in the study of pronoun interpretation. We employed the eye-tracking method within the visual world paradigm wherein the eye-gazes to the pictures reflect pronoun resolution during online processing. Yet, this method does not only provide insight into the different sources of information considered during online comprehension but is also an implicit measure of sentence comprehension which reduces task demands especially for children (e.g., Brandt-Kobele and Höhle, 2010; Bergmann et al., 2012). However, the technique also has its limitations. The online results may not always correspond to offline responses (Schumacher et al., 2017) because they do not capture processes during later stages of sentence processing/interpretation. Furthermore, the method may be more time-consuming compared to offline methods regarding to the creation of the experimental materials (visual and auditory material) and the preprocessing and the analyses of eye-movements.

In addition, our study underlines that research on pronoun resolution (or more general language use/production and comprehension) should consider both linguistic and psycholinguistic approaches. In particular, our study demonstrates that, in addition to the linguistic factors (e.g., agreement, personal pronoun vs. d-pronoun), processing factors like grammatical role parallelism influence pronoun resolution. In this way, it emphasizes the requirement that linguistic theories should be based on empirical work that employs different methods.

## Conclusion

To summarize, we reported on the first study comparing the impact of word order and parallelism effects on online pronoun resolution in German. We showed that parallelism of the grammatical role had an early and strong effect on the processing of the pronoun, regardless of the word order of the antecedents. This suggests that different sources of information are considered during online pronoun resolution (cf., Arnold et al., 2000; Kehler et al., 2008; Schumacher et al., 2016, 2017) and that parallelism is one of the crucial factors in this process (cf., Smyth, 1994). In addition, our results indicate that personal pronouns may not in general be associated with the subject or topic of a sentence in German but that their resolution is modulated by additional factors such as the grammatical role. Further studies are required to investigate whether parallelism also affects offline antecedent choices and whether the parallelism may also influence pronoun resolution of demonstrative pronouns. In this way, the interaction between parallelism and information structure may be clarified.

## Author Contributions

NG was responsible for the design and procedure of the experiments. NG supervised a Ph.D.-student, Elena Valentik-Klein, who is not working in research anymore, during the creation the materials. The experiment was carried out by student researchers. AS conducted the data analyses. AS and NG co-authored the paper.

## Conflict of Interest Statement

The authors declare that the research was conducted in the absence of any commercial or financial relationships that could be construed as a potential conflict of interest.

## Footnotes

1 Note that in German determiners are also marked with respect to gender, but we did not indicate the gender in the glosses. In this example as well as in the items of our study, both the antecedents and the anaphora are masculine.

2 The models revealed an interaction between Time and Pronoun type in the second time window between 250 and 500 ms. Note, however, that it takes around 200 ms to initiate a saccade (e.g., Sumner, 2011) and around 400 ms to utter the pronouns. Accordingly, we do not expect the pronoun to have already had an effect in this time window.
