# Supplementary material for: Grammatical Role Parallelism Influences Ambiguous Pronoun Resolution in German
Source: Front Psychol. 2017 Jul 25;8:1205. doi: 10.3389/fpsyg.2017.01205 (PMC5524765; doi:10.3389/fpsyg.2017.01205)
Supplement: Supplementary file 1 [file Data_Sheet_1.DOC]

**Supplementary material**

A1) Der Bulle und der Elefant spielen zusammen Verstecken im Wald.

“The bull and the elephant are playing hide and seek together in the forest.”

Der Bulle sieht den Elefanten. Er… ist traurig. / Ihn… trifft der Blitz.

“The bull sees the elephant. He… is sad. / Him… the lightning hits.”

A2) Der Bär und der Wolf suchen einander in einem Park.

“The bear and the wolf are looking for each other in a park.”

Der Bär ruft den Wolf. Er… mag den Frühling. / Ihn… irritiert der Wind.

“The bear calls the wolf. He… likes the spring. / Him… the wind bothers.”

A3) Der Tiger und der Löwe kaufen für einander Farben in einem Geschäft.

“The tiger and the lion are buying paints for each other in a shop.”

Der Tiger malt den Löwen. Er... ist nervös. / Ihn... quält die Hitze.

“The tiger draws the lion. He... is nervous. / Him... the heat tortures.”

A4) Der Hase und der Biber rennen hintereinander auf einer Wiese.

“The hare and the beaver are running after each other in a meadow.”

Der Hase holt den Biber ein. Er... regt sich auf. / Ihn... erfreut das schöne Wetter.

“The hare catches up with the beaver. He ... is upset. / Him ... the nice weather pleases.”
